# A Case Suggestive of an Aorto-Esophageal Fistula Caused by Pseudoaneurysm at the Distal Anastomosis of an Aortic Graft: Long-Term Survival without Aortic Reconstruction

**DOI:** 10.70352/scrj.cr.25-0266

**Published:** 2025-09-26

**Authors:** Kazuki Noda, Hiromichi Fujii, Shigeru Lee, Goki Inno, Takumi Kawase, Yukihiro Nishimoto, Munehide Nagao, Ryo Nangoya, Yosuke Takahashi

**Affiliations:** 1 Department of Cardiovascular Surgery, Osaka Metropolitan University, Osaka, Japan; 2 Department of Gastroenterological Surgery, Osaka Metropolitan University, Osaka, Japan

**Keywords:** aorto-esophageal fistula, thoracic endovascular aortic repair, esophagectomy

## Abstract

**INTRODUCTION:**

Aorto-esophageal fistula (AEF) is a relatively rare and life-threatening condition. Although various treatment strategies have been developed, including conventional surgical repair, long-term survival remains uncommon. This report presents a case of AEF successfully treated without aortic reconstruction, resulting in long-term survival.

**CASE PRESENTATION:**

The patient was a 74-year-old male with a medical history of total arch replacement and severe emphysema, who presented to our department with hematemesis. Upper gastrointestinal endoscopy results revealed an AEF caused by the prosthetic graft. Emergency thoracic endovascular aortic repair was performed, followed by urgent subtotal esophagectomy the next day, without aortic reconstruction due to the patient’s frailty. The patient underwent gastric conduit reconstruction via the presternal route 7 months later and has remained uneventful for over 6 years.

**CONCLUSIONS:**

The treatment without aortic reconstruction combined with conservative treatment for AEF might be feasible for high-risk surgical patients.

## Abbreviations

AEF
: aorto-esophageal fistula
TEVAR
: thoracic endovascular aortic repair

## INTRODUCTION

AEF is a relatively rare and life-threatening condition.^1)^ One of the major causes of AEF is a complication following surgical prosthetic repair of an aortic aneurysm or TEVAR.^1)^ Conservative treatment generally results in poor long-term outcomes,^2)^ whereas an aggressive approach, including concomitant esophageal, aortic, and stent graft resection, aortic reconstruction, and omentopexy, may provide a better outcome in a previous report.^3)^ The management of AEF remains difficult in controlling the associated infection.^4)^ Furthermore, this study presents the case of an AEF treated without aortic reconstruction for a high-risk surgical patient, resulting in long-term survival.

## CASE PRESENTATION

**Fig. 1:**
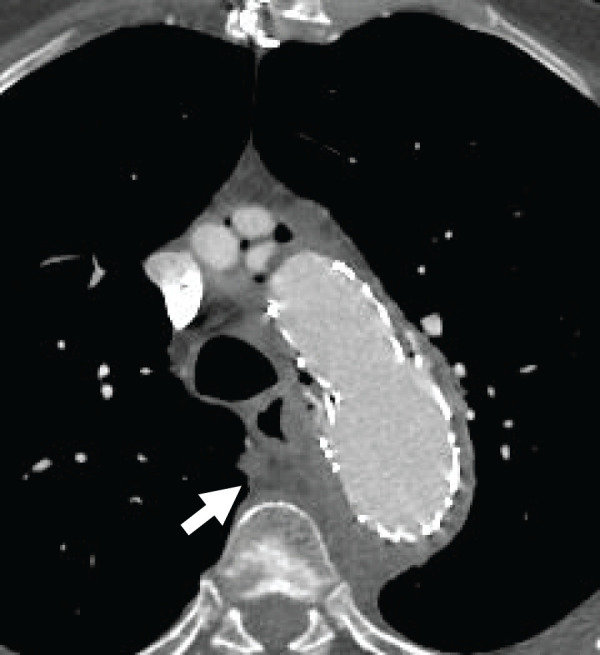
The CT results revealed free air surrounding the prosthetic graft and the collection of fluid resembling an abscess around the esophagus (white arrow).

**Fig. 2:**
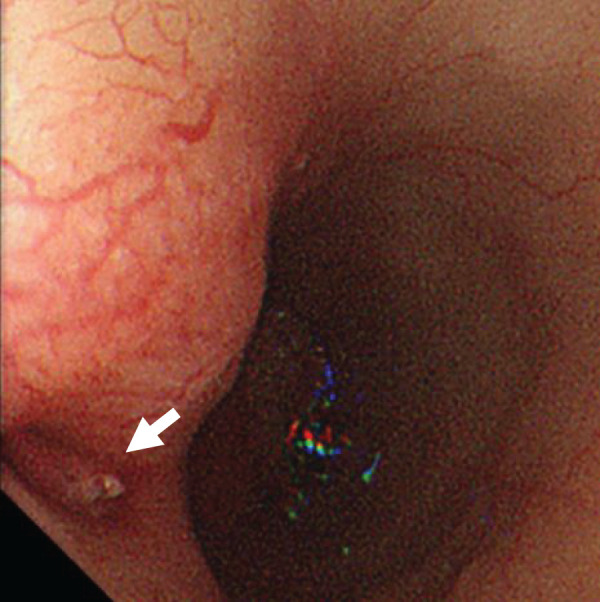
The upper gastrointestinal endoscopy results revealed an esophageal ulcer near the distal anastomosis site (white arrow).

A 74-year-old male presented to our hospital with hematemesis. His medical history included a total arch replacement for a thoracic aortic aneurysm 1 year ago, TEVAR for pseudoaneurysm at the distal anastomosis site 4 months ago, partial resection of the right lower lung lobe for lung adenocarcinoma 2 months ago, and severe emphysema. On arrival, his blood pressure was 123/61 mm Hg and pulse rate was 113 beats/min. The CT results revealed free air around the aortic arch with the prosthetic graft, along with a fluid collection suggestive of an abscess near the esophagus (**Fig. 1**). Laboratory findings showed elevated white blood cell count and C-reactive protein levels (**Table 1**). After hospitalization, the blood cultures were obtained, and empirical broad-spectrum antibiotic therapy was initiated with intravenous meropenem (1 g every 12 hours) and vancomycin, administered under therapeutic drug monitoring. The next day, he experienced recurrent massive hematemesis. Urgent upper gastrointestinal endoscopy revealed an esophageal ulcer adjacent to the distal anastomosis site, raising suspicion of AEF (**Fig. 2**). Because of persistent hematemesis and his preshock state, emergency TEVAR was prioritized and performed on the same day to control the hematemesis and stabilize the hemodynamics. Although extravasation was not observed via angiography, a stent graft was deployed for reinforcement of the previous stent graft, which covered the pseudoaneurysm at the distal anastomosis site. On the following day, esophagectomy, prosthetic graft removal, and thoracic aortic reconstruction were considered as curative procedures. However, after a multidisciplinary discussion between the cardiovascular and esophageal surgery teams, an objective risk assessment using EuroSCORE II estimated an operative mortality of 21.8%, and aortic reconstruction was deemed high risk. Also, given his frailty, severe emphysema, and history of post-lobectomy, which made prolonged one-lung ventilation unfeasible, the surgical strategy was modified. Therefore, esophagectomy alone was performed.

**Table 1:** Laboratory data at admission

Variables	At admission	Normal range
White blood cell count (*10^2^/μL)	168	43~80
Hemoglobin (g/dL)	11.6	12.4~17.2
Platelet count (*10^4^/μL)	46.5	18.0~34.0
C-reactive protein (mg/dL)	22.0	<0.3
Total protein (g/dL)	6.9	6.6~8.1
Albumin (g/dL)	2.9	3.5~5.0
Blood urea nitrogen (mg/dL)	18	8~20
Creatinine (mg/dL)	0.9	0.5~1.1
Sodium (mmol/L)	137	138~145
Potassium (mmol/L)	3.7	3.6~4.8
Chlorine (mmol/L)	100	101~108
Total Bilirubin (mg/dL)	0.4	0.2~1.0
Aspartate aminotransferase (U/L)	12	13~30
Alanine aminotransferase (U/L)	11	8~42
Alkaline phosphatase (U/L)	310	38~113
Gamma-glutamyl transpeptidase (U/L)	36	13~64
Amylase (U/L)	29	44~132
Creatine kinase (U/L)	35	59~248
Lactate dehydrogenase (U/L)	125	124~222
Prothrombin time (%)	71	70~130
Activated partial thromboplastin time (sec)	36.2	24.0~34.0
Fibrinogen (mg/dL)	506	200~400
Fibrinogen/fibrin degradation products (μg/mL)	4.4	<5.0
Thyroid-stimulating hormone (μIU/mL)	2.58	0.50~5.00
Free thyroxine (ng/dL)	1.47	0.90~1.70
Procalcitonin (ng/mL)	0.36	<0.046

### Surgical procedure

**Fig. 3:**
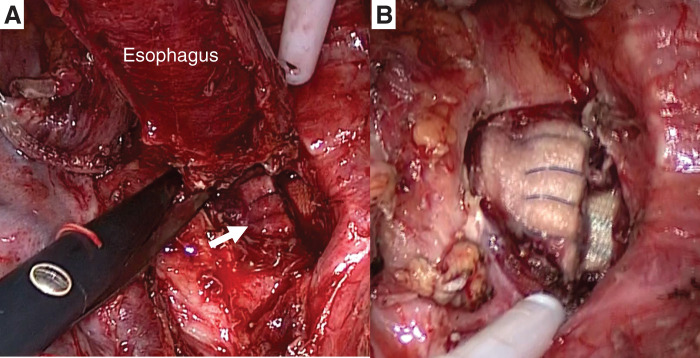
After resection of the aorto-esophageal fistula, the prosthetic graft and felt strip (white arrow) were revealed (**A**). The abscess around the fistula was removed (**B**).

**Fig. 4:**
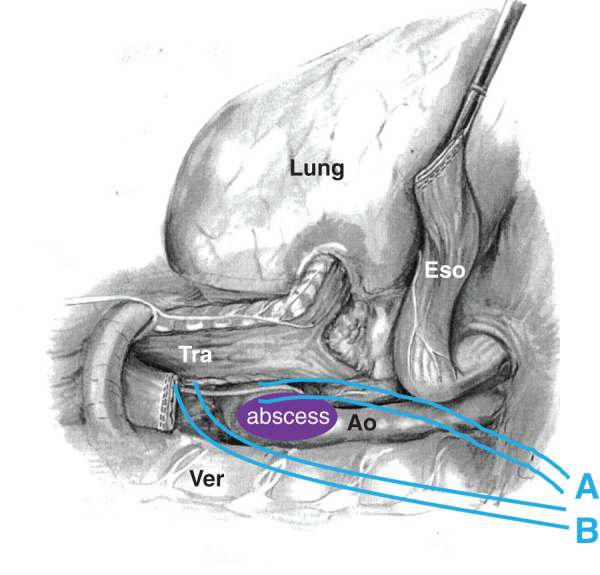
The schema of the drainage tubes. A double-lumen tube was placed alongside the abscess (**A**), while a drainage tube was positioned on the dorsal side of the abscess (**B**). Ao, aorta; Eso, esophagus; Tra, trachea; Ver, vertebra

The patient was placed in the left decubitus position, and an endobronchial blocker was inserted through a single-lumen endotracheal tube. A right posterolateral thoracotomy was performed at the 5th intercostal space. Upon cranial resection of the esophagus, the prosthetic graft and felt strip used at the distal anastomosis were exposed (**Fig. 3A**). Based on these findings, the diagnosis of AEF secondary to pseudoaneurysm formation at the distal anastomosis was confirmed. Following subtotal esophagectomy, the abscess around the fistula was removed (**Fig. 3B**). The right thoracic cavity was irrigated with saline, followed by the application of gentian violet. A double-lumen tube was placed alongside the prosthetic graft for irrigation, and a drainage tube was positioned on the dorsal side (**Fig. 4**). Furthermore, cervical esophagostomy and enterostomy were performed. After meticulous hemostasis, the wound was closed in layers.

### Postoperative course

**Fig. 5:**
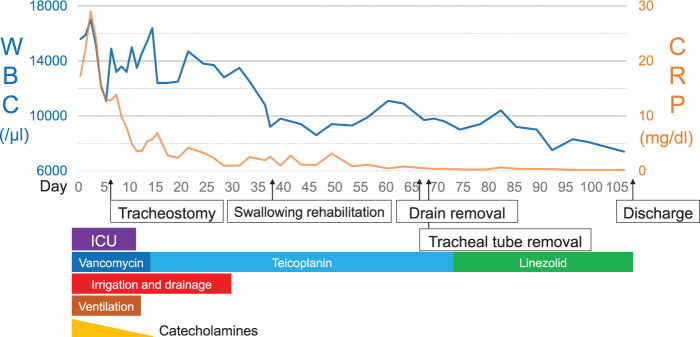
The clinical course of the patient. After the operation, continuous saline irrigation and antibiotics with vancomycin were initiated. The inflammatory response reduced gradually, and the catecholamines were tapered to discontinuation. The patient had recovered enough to be transferred to a general ward; the drainage tubes were removed, and the antibiotics were switched to oral antibiotics with linezolid. No further relapse was observed, and the patient was discharged on day 108. CRP, C-reactive protein; WBC, white blood cell

The patient’s postoperative clinical course is shown in **Fig. 5**. On day 1, the preoperative blood cultures revealed methicillin-resistant coagulase-negative *Staphylococcus* spp., and continuous saline irrigation with 1 L of saline per day from the double-lumen tube was initiated. On day 6, he underwent a tracheostomy due to respiratory failure. On day 14, the antibiotic was changed from vancomycin to teicoplanin because of renal dysfunction. On day 28, the continuous saline irrigation was discontinued. The drain tube was replaced with a smaller-sized tube after internal fistulization and was gradually withdrawn and finally removed on day 67. The tracheostomy tube was removed on day 68, and the antibiotics were switched to oral antibiotics (600 mg of linezolid given orally every 12 h) on day 73. The CT results revealed no findings of free air in the mediastinum on day 97, indicating its complete resolution. After rehabilitation, he was discharged home on day 108. He underwent gastric conduit reconstruction via the presternal route 7 months later, which has remained uneventful and has been receiving oral antibiotic prophylaxis under outpatient follow-up for over 6.5 years. Annual CT scans and routine monitoring of inflammatory markers have shown no signs of recurrence or inflammation.

## DISCUSSION

AEF secondary to thoracic aortic aneurysm repair is a rare and fatal condition.^1,5)^ Its optimal treatment remains controversial due to limited studies, although the optimal strategy depends on the individual patient’s condition.^6)^

Previous reports have shown that conservative medical management is strongly associated with in-hospital mortality.^7)^ Considering that infection control is important for long-term survival,^3)^ a definitive treatment such as aortic and esophageal reconstruction was crucial for the source control of the prosthetic graft in the present case. The standard surgical approach for AEF repair usually involves a left thoracotomy with cardiopulmonary bypass, with or without deep hypothermic circulatory arrest.^8)^ However, in this case the patient had previously undergone significant challenges such as total arch replacement, which would have required complete removal of the previously implanted grafts, esophagectomy, and reconstruction from the ascending aorta to the descending aorta, which significantly increased surgical invasiveness. Considering the patient’s medical history of right partial lobectomy, severe emphysema, and marked frailty, performing these procedures in a single stage was deemed unfeasible. Additionally, the extra-anatomical bypass was not considered for cases involving high morbidity and the risk of further aortic rupture.^9)^ Thus, esophagectomy was performed to prevent contamination from the esophagus, and it was considered better in a staged approach without immediate aortic reconstruction. In practice, infection control was achieved through continuous irrigation and drainage, along with long-term antibiotic therapy after esophagectomy. Importantly, recent literature emphasizes that although open surgery remains the gold standard, individualized approaches are essential. For high-risk or frail patients, less invasive or staged treatments, such as TEVAR combined with esophageal stenting^10)^ or esophageal preservation using flap coverage with omentum instead of esophagectomy,^11)^ have been reported to achieve acceptable outcomes. However, conservative treatment without any intervention is consistently associated with poor prognosis and is generally considered only in palliative settings.^12)^ In this case, the combination of esophagectomy without aortic reconstruction was proven to be the most favorable treatment approach in terms of prognosis and infection control.

As a conservative method, the effectiveness of saline irrigation and tube drainage may be argued in this case. Generally, if an abscess cavity is inappropriately irrigated or lavaged, the abscess could spread unless an absolute drainage is performed. In previous reports, this strategy could be feasible and effective to minimize bacterium counts and promote cavity narrowing^13,14)^ in several cases. However, the maneuvers, including the irrigation volume and speed, have not been established and mostly depend on individual judgments. In this case report, to ensure safety, a drainage tube was placed in each cavity and dorsal side (**Fig. 4**), whereas 1 L of saline per day was initiated and the volume was manually adjusted while monitoring the drainage output. Additionally, as part of the infection control strategy, gentian violet was applied locally to the contaminated area after esophagectomy. Despite its known carcinogenic potential,^15)^ its use in this case was based on published clinical precedents^3,16)^ and the potential benefit in infection control during a life-threatening situation. The successful reduction of bacterial counts through these maneuvers can be attributed to the success of conservative treatment after surgery.

## CONCLUSIONS

This study reported a successful surgical case of an AEF treated without aortic reconstruction. In patients with frailty and who are unsuitable for open surgery, TEVAR and total esophagectomy as a staged treatment may be effective when successfully combined with conservative methods.

## DECLARATIONS

### Availability of data and materials

The datasets used and/or analyzed during the current study are available from the corresponding author on reasonable request.
